# Concurrent central diabetes insipidus and cerebral salt wasting disease in a post-operative case of craniopharyngioma: a case report

**DOI:** 10.1186/s12887-021-02982-9

**Published:** 2021-11-10

**Authors:** Patel Zeeshan Jameel, Sham Lohiya, Keta Vagha, Tauheed Ahmed, Divya Pujari, Jayant Vagha, Ashish Varma

**Affiliations:** 1 Department of Paediatrics, Jawaharlal Nehru Medical College, Sawangi (Meghe), Wardha, Maharashtra 442001 India; 2 Department of Medicine, Tata Main Hospital, Jamshedpur, Jharkhand India; 3 Division of Paediatric Endocrinology, Department of Paediatrics, Bai Jerbai Wadia Hospital for Children, Mumbai, Maharashtra India

**Keywords:** Central diabetes insipidus, Cerebral salt wasting disease, Craniopharyngioma

## Abstract

**Background:**

Water and electrolyte disorders commonly encountered in children post-surgery involving hypothalamus and posterior pituitary, are central diabetes insipidus, syndrome of inappropriate secretion of anti-diuretic hormone and cerebral salt wasting disease. Delayed diagnosis and inadequate management of such cases may lead to worsened neurological outcomes with a high mortality rate.

**Case presentation:**

Here we report the case of a 7-year-old girl who underwent surgical resection of a craniopharyngioma, following which she initially developed central diabetes insipidus. However, later on in the course of her illness she developed symptomatic hyponatremia with natriuresis which was diagnosed to be due to cerebral salt wasting disease. This combination of central diabetes insipidus and cerebral salt wasting syndrome is a rare occurrence and poses a diagnostic challenge. Diagnosis and management can be even more difficult when these conditions precede or coexist with each other.

**Conclusion:**

In such cases development of hyponatremia should always prompt consideration of unusual causes like cerebral salt wasting disease in addition to the classically described syndrome of inappropriate secretion of anti-diuretic hormone. Hence, a thorough knowledge of these disorders along with intensive monitoring of fluid and sodium status is critical for timely diagnosis and management of these patients.

## Background

Fluid and electrolyte imbalance is commonly encountered in children secondary to central nervous system (CNS) insult. Among children undergoing surgeries in the sellar and suprasellar regions, most commonly due to craniopharyngioma, the incidence of fluid and electrolyte disorders is quite high. Central diabetes insipidus (CDI), syndrome of inappropriate antidiuretic hormone secretion (SIADH), cerebral salt wasting (CSW) disease and adipsic diabetes insipidus (ADI) can occur individually or exist simultaneously making their diagnosis and management extremely troublesome.

Here, we present the case of a female child with CDI and CSW disease occurring simultaneously after surgical resection of craniopharyngioma.

## Case presentation

**Fig. 1:**
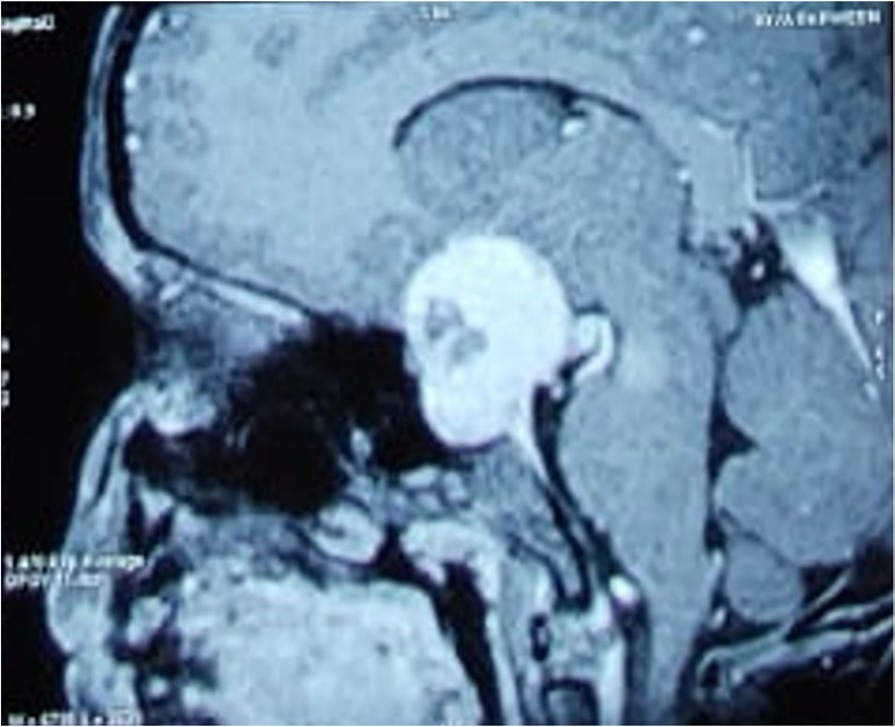
T1W image showing iso to hyperintense mass involving the sella and suprasellar region

A 7-year-old girl presented with complaints of headache for the past 1 month and progressive right sided weakness for the past 15 days. On examination, the child had right sided hemiplegia, bilateral hemianopia and signs of optic atrophy in both eyes. MRI brain showed a 3x2x0.5 cm large sellar and supra-sellar mass with T1 weighted image showing iso to hyperintense lesion suggestive of a craniopharyngioma (Fig. 1). Pre-operative hormonal levels (GH, TSH, free T4, 8 AM cortisol, FSH, LH and prolactin) were within normal limits. She underwent gross total resection of the tumor by left fronto-temporal craniotomy. As the tumor was infiltrating the pituitary stalk, it had to be sacrificed along with the infundibulum.

Following surgery, she developed polyuria and her urine output was 8 ml/kg/hr. Further investigations showed elevated serum sodium (156 mEq/L), raised plasma osmolality (321 mOsm/kg) and decreased urinary osmolality (175 mOsm/kg). Her blood sugars were within normal limits (122-140 mg/dL). She was diagnosed to be suffering from CDI. Fluid losses in excess of the normal maintenance fluid rate were replaced volume for volume with 0.9% normal saline 6 hourly. She was started on IV dexamethasone (0.2 mg/kg/day; 6 hourly) and subcutaneous vasopressin (4 IU/day; 6 hourly). Urine output and serum sodium levels were strictly monitored. She also complained of no light perception in both of her eyes. On the second post-operative day, she continued to have polyuria although a decrease was observed from the previous day (7 ml/kg/hr). Sodium levels increased to 160 mEq/L. The dose of vasopressin was increased further (6 IU/day) and administered when required after she had episodes of breakthrough polyuria. By the third post-operative day, her urine output had stabilized at around 3-4 ml/kg/hr. and her sodium levels were between 136 and 143 mEq/L. Her subcutaneous dose was changed to oral desmopressin (0.1 mg/day) twice daily. Oral dosage was also regulated as per her urine output with daily monitoring of serum sodium levels. On the seventh post-operative day, hormonal profile was suggestive of hypocortisolism (8 AM plasma cortisol: 1.47 μg/dL) and central hypothyroidism (TSH: 0.44 mIU/L, Free T4: 0.44 ng/dL). She was started on levothyroxine 25 mcg. In addition, as the dexamethasone was stopped by fifth post-operative day, she was started on hydrocortisone 1.25 mg (10 mg/m^2^/day) 6 hourly.

**Fig. 2:**
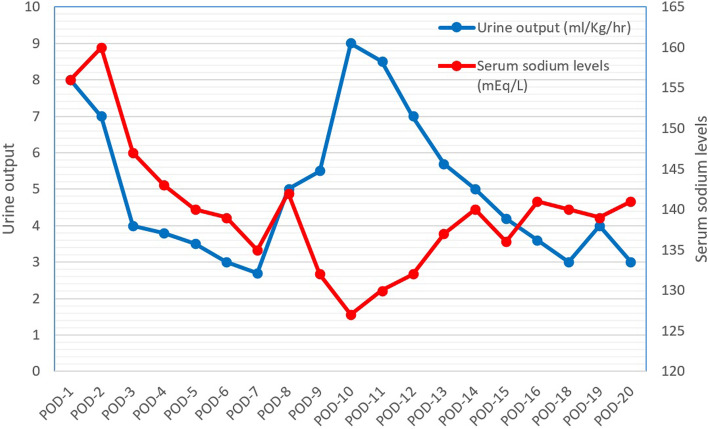
Trend of urine output (*blue*) and serum sodium levels (*red*) through the post-op period. Initially, a phase of diuresis [U/O: 8 ml/kg/hr.; Sr Na^+^: 156 mEq/L] was followed by a phase of natriuresis and hyponatremia by the 10th post-operative day due to CSW disease [U/O: 9 ml/kg/hr.; Sr Na^+^: 127 mEq/L]. The final stage was permanent diabetes insipidus after waning of CSW disease. POD: Post-operative day, U/O: Urine output; Sr Na^+^: serum sodium level

On the ninth post-operative day, her mother noticed polyuria and irritability. In view of her polyuria, increasing the dose of desmopressin further was considered but was not done as the serum sodium was 132 mEq/L. Fluid losses in excess of the normal maintenance fluid rate was replaced volume for volume with 0.9% normal saline 6 hourly. However, there was no relief in her symptoms. In addition, on the tenth post-operative day, she was became increasingly drowsy. Her repeat serum sodium was 127 mEq/L. There was a rise in hemoglobin and hematocrit as well. Desmopressin was withheld. A paired plasma osmolality was 261 mOsm/kg and urinary osmolality was 615 mOsm/kg. Her urinary sodium was 218 mEq/L despite having hyponatremia. Repeat serum urea, BUN, serum creatinine and serum potassium were within normal limits for her age. Her blood glucose was also normal. A diagnosis of CSW was thus made. A single bolus of hypertonic saline (3% NaCl) was administered over 20-30 min followed by a continuous infusion in view of symptomatic hyponatremia. Tab fludrocortisone was started at 0.1 mg/day. Hypertonic saline infusion was tapered and stopped after 12 h as sodium levels normalized. Over the next 2 days, serum osmolality (292 mOsm/kg) and urinary osmolality (300 mOsm/kg) had normalized along with a reduction in urinary sodium level (25 mEq/L). Her sensorium had improved but polyuria persisted. Desmopressin was restarted (0.1 mg/day) once the sodium level had normalized as polyuria was due to CDI. Given the above clinical scenario, an overlap of CDI and CSW disease was considered in the present case. Figure 2 summarizes the changes in urine output and serum sodium levels throughout the period of her hospital stay. Over the next couple of days, her desmopressin dose was adjusted and fludrocortisone was reduced to 0.05 mg/day. Tab. Fludrocortisone was later tapered and stopped after 7 days. She was discharged after 20 days of hospital stay on hydrocortisone, levothyroxine and desmopressin.

On follow-up after 2 weeks, her hormonal profile showed low TSH (0.52 mIU/L), normal free T4 (1.22 ng/dL) and low 8 AM cortisol (4.1 mcg/dL) requiring increase in dosages of levothyroxine to 37.5 mcg/ day and tab. Hydrocortisone to 2.5 mg (10 mg/m^2^/day) 8 hourly. Serum sodium and potassium levels were normal for her age. After 2 weeks, a repeat hormonal profile (TSH, free T4 and 8 AM cortisol) was within normal limits. She is now clinically well with her permanent DI under control on 0.15 mg/day of desmopressin. As part of her neurological morbidity, she has hemiparesis with no improvement in power and has only light perception in both eyes.

## Discussion and conclusion

Central nervous system (CNS) performs a crucial role in the maintenance of water and sodium homeostasis resulting from a complex interplay between hypothalamus, posterior pituitary, and adrenal glands. Factors disrupting the regulation of this normal HPA axis result in pathological conditions such as CDI, SIADH and CSW disease. Craniopharyngiomas account for 6-9% of brain tumors in children [1]. Endocrine dysfunction is a well-known complication arising from surgical or radiation treatment of craniopharyngioma [1]. Children were found to be particularly vulnerable to pituitary hormonal deficiency post-surgery. Incidence of a single or multiple pituitary hormone deficiency can range from 36 to 98% after surgery [2–6]. In addition to this, the incidence of water and sodium related disorders in children undergoing surgeries involving sellar and supra-sellar region varies from 40 to 72% [7, 8].

Post-pituitary surgery hyponatremia is commonly encountered and carries a wide spectrum of differential diagnosis such as improper fluid administration, hypocortisolism, hypothyroidism, over-correction of DI in over-dosing of desmopressin, SIADH and CSW disease. Hypocortisolism may lead to hyponatremia by causing an increase in ADH levels along with impaired renal free water excretion [7, 9]. To prevent this hydrocortisone should be started at 10-12 mg/m^2^/kg (in 3-4 divided doses) preferably pre-operatively although a steroid sparing approach may also be used where steroids are only started when hypocortisolism is present [10]. In cases where the child is already on dexamethasone, no additional hydrocortisone is required. Post-surgery, dexamethasone should be carefully tapered along with a parallel increase in dose of hydrocortisone.

Central diabetes insipidus (CDI) results from a deficiency of arginine vasopressin (AVP), and is characterized clinically by polyuria, polydipsia, and dehydration [11–13]. CDI has been reported in upto 80% of children post-surgery [13]. Pathologic polyuria (> 4 ml/kg/hr) with raised serum osmolality (> 300 mOsm/kg) and hypernatremia (> 145 mEq/L) but decreased urine osmolality (< 300 mOsm/kg) with low urine specific gravity (< 1.010) establishes a diagnosis of CDI [11–13]. It is essential to rule out hyperglycemia and cortisol deficiency prior to establishing a diagnosis of CDI. Injury to the pituitary stalk during surgery or head injury can lead to a transient, triphasic or permanent CDI. Transient CDI can manifest within hours of surgery with polyuria lasting for 5-7 days. It usually settles once the AVP secreting neurons located in the posterior pituitary recover their normal function. In the triphasic response, the first phase of transient CDI is followed by second phase of SIADH resulting from an unregulated release of ADH from the dying or damaged axons in posterior pituitary. SIADH is characterized by oliguria, decreased serum osmolality (< 280 mOsm/kg), hyponatremia (< 135 mEq/L), urine osmolality (> 100 mOsm/kg) and urine sodium excretion of > 30 mEq/L [14, 15]. Usually, after about 2 weeks, re-emergence of polyuria marks the onset of third phase of permanent CDI resulting from the death of ADH secreting neurons [16].

In addition to these presentations, rarely patient might develop CSW disease due to a primary neuronal insult or as a secondary response to SIADH [16]. CSW disease has been hypothesized to result either from increased levels of natriuretic peptides, especially BNP, or due to a poor sympathetic outflow to kidneys [15]. CSW disease was first described by Peters et al. in 3 patients with neurological disorder and hyponatremia [15, 17]. However, for the following 20 years, CSW disease was an under-recognized entity until Nelson et al. [18] conclusively demonstrated volume depletion and hyponatremia with increased urinary sodium excretion consistent with CSW disease pathogenesis. CSW disease can occur secondary to any form of CNS insult such as head trauma, stroke, subarachnoid hemorrhage, hydrocephalus, TB meningitis, or even post-operatively after resection of brain tumors [19–21]. Diagnosis of an underlying CSW disease in children can be established in the presence of elevated urinary sodium excretion (> 120 mEq/L) and urine osmolality (> 300 mOsm/kg) despite the child having polyuria (≥3-4 ml/kg/hr), dehydration, hyponatremia (< 130 mEq/L) and a negative 24 h fluid balance [19, 20]. Incidence of CSW disease in post-operative cases of brain tumor among children has been observed to be more common than SIADH, contrary to previous belief. González Briceño L et al. [7] found CSW disease to be more common (3.8% vs 1.3%) than SIADH on follow-up post-surgery of suprasellar tumors in children. Similarly, Hardesty DA et al. [22] also observed CSW disease in 5% of post-op children as compared to 3% of SIADH cases. Another study by Williams CN et al. [23] observed CSW disease more common than SIADH (44% vs 26%) in post-op cases of brain tumor. In addition, identified risk factors include younger age (≤ 7 years) and female sex [23]. SIADH and CSW disease both have similar biochemical laboratory parameters such as low serum osmolality, high urine osmolality and natriuresis. Despite the similarities, natriuresis is considerably higher than the sodium intake in CSW while it is more or less equal in SIADH. In addition to this, SIADH patients have a state of euvolemia or mild extracellular fluid expansion while patients CSW disease are dehydrated with a negative fluid balance due to severe natriuresis [16]. Both SIADH and CSW disease present with post-operative hyponatremia. In all such cases, hypocortisolism and hypothyroidism need to be excluded. An early peri-operative glucocorticoid replacement, in addition to preventing/correcting hyponatremia, also helps to reduce cerebral edema.

The coexistence of CDI and CSW disease is extremely rare, rather CDI is associated with SIADH especially in operated patients of sellar and supra-sellar tumors. Combined CDI and CSW disease has been reported in patients suffering from traumatic brain injury, cardiac arrest, cardiogenic and septic shock, meningitis, encephalitis, shaken baby syndrome, intracranial hemorrhage, sellar and/or supra-sellar tumors [19, 24–27]. Prognosis in all of these cases was found to variable and dependent upon the underlying cause as well as effective treatment of both CDI and CSW disease. In our case, child had initially developed CDI, however, in stark contrast to the classically described ‘triphasic response’, our patient developed CSW disease after an initial phase of CDI. SIADH and CSW disease can be particularly difficult to differentiate as both present with hyponatremia. The measurement of fluid input, urine output, serum osmolality, and urine osmolality helps to differentiate CSW disease from SIADH. In our case as well, increased urinary osmolality and increased urinary sodium excretion despite the child having hyponatremia, dehydration and decreased serum osmolality guided us to consider CSW disease over SIADH. CSW disease was a transient event as it responded quite well to fludrocortisone and ultimately the drug was stopped. Later on, she was maintained on levothyroxine, hydrocortisone and desmopressin for managing her hypothyroidism, hypocortisolism and diabetes insipidus.

To conclude, in cases of CDI following surgery, development of hyponatremia should always prompt consideration of unusual causes like CSW in addition to the classically described SIADH. Early recognition and differentiation of CSW disease from SIADH is important, as management strategies for these conditions are completely different, and a misdiagnosis or even a delay in diagnosis can prove fatal. Prompt diagnosis and appropriate management of CDI and CSW disease was extremely crucial in the survival of our patient. We therefore recommend a close watch on serum sodium, and urine output in all operated cases of sellar/suprasellar tumors, with a low threshold for measuring urinary sodium excretion, urine osmolality and serum osmolality.

## Learning points

- A triphasic response in not necessarily the rule of thumb in operated cases involving the pituitary stalk.
- A greater emphasis is required on the importance of close monitoring after pituitary stalk injury post-surgery to prevent further endocrine morbidity and mortality.

## Data Availability

The datasets used and/or analyzed during the current study are available from the corresponding author on reasonable request.

## Abbreviations

CNS: Central nervous system
CDI: Central diabetes insipidus
CSW: Cerebral salt wasting
SIADH: Syndrome of inappropriate antidiuretic hormone secretion
MRI: Magnetic resonance imaging
GH: Growth hormone
TSH: Thyroid stimulating hormone
FSH: Follicle stimulating hormone
LH: Luteinizing hormone
HPA: Hypothalamo-pituitary-adrenal
AVP: Arginine vasopressin
